# X-ray dark-field imaging of the human lung—A feasibility study on a deceased body

**DOI:** 10.1371/journal.pone.0204565

**Published:** 2018-09-27

**Authors:** Konstantin Willer, Alexander A. Fingerle, Lukas B. Gromann, Fabio De Marco, Julia Herzen, Klaus Achterhold, Bernhard Gleich, Daniela Muenzel, Kai Scherer, Martin Renz, Bernhard Renger, Felix Kopp, Fabian Kriner, Florian Fischer, Christian Braun, Sigrid Auweter, Katharina Hellbach, Maximilian F. Reiser, Tobias Schroeter, Juergen Mohr, Andre Yaroshenko, Hanns-Ingo Maack, Thomas Pralow, Hendrik van der Heijden, Roland Proksa, Thomas Koehler, Nataly Wieberneit, Karsten Rindt, Ernst J. Rummeny, Franz Pfeiffer, Peter B. Noël

**Affiliations:** 1 Department of Physics and Munich School of BioEngineering, Technical University of Munich, Garching, Germany; 2 Department of Diagnostic and Interventional Radiology, Klinikum rechts der Isar, Technical University of Munich, Munich, Germany; 3 Institute of Forensic Medicine, Ludwig-Maximilians-University Munich, Munich, Germany; 4 Institute of Clinical Radiology, Ludwig-Maximilians-University Munich, Munich, Germany; 5 Karlsruhe Institute of Technology, Institute of Microstructure Technology, Eggenstein-Leopoldshafen, Germany; 6 Philips Medical Systems DMC GmbH, Hamburg, Germany; 7 Philips GmbH Innovative Technologies, Research Laboratories, Hamburg, Germany; 8 Institute for Advanced Study, Technical University of Munich, Garching, Germany; New York University School of Medicine, UNITED STATES

## Abstract

Disorders of the lungs such as chronic obstructive pulmonary disease (COPD) are a major cause of chronic morbidity and mortality and the third leading cause of death in the world. The absence of sensitive diagnostic tests for early disease stages of COPD results in under-diagnosis of this treatable disease in an estimated 60–85% of the patients. In recent years a grating-based approach to X-ray dark-field contrast imaging has shown to be very sensitive for the detection and quantification of pulmonary emphysema in small animal models. However, translation of this technique to imaging systems suitable for humans remains challenging and has not yet been reported. In this manuscript, we present the first X-ray dark-field images of *in-situ* human lungs in a deceased body, demonstrating the feasibility of X-ray dark-field chest radiography on a human scale. Results were correlated with findings of computed tomography imaging and autopsy. The performance of the experimental radiography setup allows acquisition of multi-contrast chest X-ray images within clinical boundary conditions, including radiation dose. Upcoming clinical studies will have to demonstrate that this technology has the potential to improve early diagnosis of COPD and pulmonary diseases in general.

## Introduction

Lung diseases, such as chronic obstructive pulmonary disease (COPD), lower respiratory infections and lung cancer are some of the most common medical conditions in the world. According to the most recent (2015) world health organization (WHO) data [1], COPD ranks level with lower respiratory infections as the third leading cause of death globally, claiming over 3 million lives each year (5.7% of all deaths). At the same time, early diagnosis of COPD is challenging, as spirometry is highly dependent on the patient’s cooperation and conventional chest radiographs have poor to moderate sensitivity. As treatment options for late stage COPD are limited, it is of major importance to improve diagnostics of COPD and lung diseases in general.

Since its clinical introduction in the early 1900s, the principle of image contrast formation in conventional X-ray imaging has solely been based on X-ray attenuation. Due to similar tissue properties in terms of the interaction mechanism with X-rays, soft-tissue contrast is low in conventional radiography (and computed tomography) and limits diagnostic capabilities. However, attenuation is not the only physical effect X-rays experience when travelling through an object. Additionally, X-rays have wave properties and are thus subject to effects like refraction and ultra small-angle scattering (which leads to dark-field contrast). Those effects cannot be visualized with a conventional X-ray imaging system. Different approaches have been developed to measure these material- and structure-specific properties and therewith provide complementary information to conventional X-ray images [2–4]. A recently developed technique that is based on a three grating interferometer arrangement [5–7] appears to be particularly promising, as its implementation is feasible with any X-ray source and detector typically used in clinical environments. The method has already been implemented in several experimental setups [8,9], a prototypical pre-clinical mammography demonstrator system [10], a radiographic projection system for osteoarthritis diagnosis in finger joints [11], and a first *in-vivo* dark-field small-animal CT system[12].

**Fig 1:**
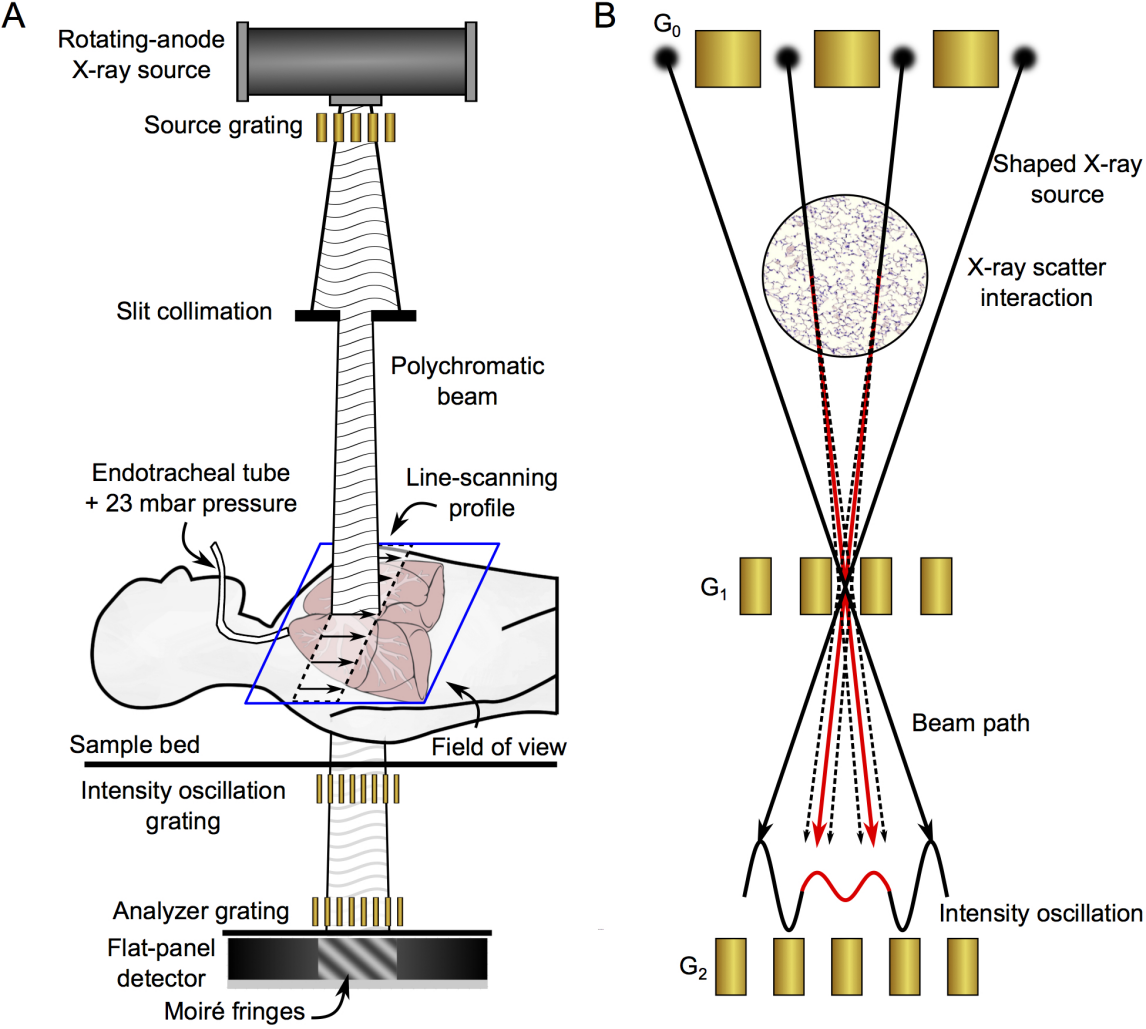
Human X-ray dark-field prototype chest scanner. (A) The X-ray chest scanner combines a three-grating arrangement (with an oblong active area) with a continuous line-scanning approach, with the purpose of imaging an entire human thorax. By scanning the active area over the field of view, every pixel is sampled at various phases of the Moiré pattern, allowing retrieval of attenuation, differential-phase, and dark-field information. In order to simulate in-vivo conditions and demonstrate clinical relevance of the presented results, tracheal intubation was performed on the deceased body and the lungs were inflated at 23 mbar during measurement. (B) X-ray dark-field contrast is generated by ultra small-angle scattering for instance at air-tissue interfaces of lung alveoli. Scattering causes blurring (red) of the initially well-delimited (black) intensity pattern generated by G1 resulting in a reduction of the measured visibility. Note that several slit sources contribute to the intensity value at any specific location in front of G2. For sake of simplicity, only a few rays passing trough one G1 slot are shown for illustrational purposes.

Dark-field image contrast is generated by multiple refractions on microstructures in the object (ultra small-angle scattering), which are on a significantly smaller scale in comparison to the spatial resolution of the imaging system. With this in mind, one can imagine that the dark-field signal indirectly reveals structural information on the micrometer length scale [6,13] that is inaccessible with conventional medical X-ray systems. Particularly the lungs exhibit a strong signal, as their main morphological structures–the alveoli–have a typical size of a few tens of micrometers with many air-tissue interfaces [12]. A schematic sketch of the experimental assembly (**A**) and its basic functional principle (**B**) are displayed in **Fig 1**.

Based on the first successful pre-clinical *in-vivo* proof-of-principle experiments [12], several small-animal models of pulmonary diseases have been investigated recently to assess the diagnostic potential of the dark-field modality. In a murine *in-vivo* model X-ray dark-field radiography showed a substantial improvement over conventional attenuation-based X-ray imaging for the diagnosis of pulmonary emphysema [14]. Notably, in a follow-up study X-ray dark-field radiography could reliably visualize different stages of emphysema *in-vivo* and demonstrated higher diagnostic accuracy for early stages of emphysema than conventional attenuation-based radiography [15]. Apart from COPD imaging preclinical X-ray dark-field radiography has shown high sensitivity for the detection and/or quantification of other pulmonary diseases like pulmonary fibrosis [16], pneumothorax [17] and neonatal lung injury [18]. Based on the preclinical *in-vivo* results, it is reasonable to argue that X-ray dark-field radiography may not only improve detection of early disease stages of COPD, but may also allow monitoring and staging of disease progression, as well as therapy response evaluation by means of the additional information on microstructural changes. However, a demonstration of X-ray dark-field imaging compatible with clinical demands in terms of image quality, scan duration, field-of-view, large object penetration and radiation dose has not been reported so far due to a number of challenges. We have overcome most of these challenges and have developed an experimental prototype [19] to evaluate the wave properties of X-ray imaging for future medical diagnostic applications. The purpose of this study was to demonstrate the feasibility of human pulmonary X-ray dark-field imaging in a lately deceased body and to correlate results with computed tomography.

## Materials and methods

The experiment was conducted in accordance with the Declaration of Helsinki and was approved by the institutional review board (Ethikkommission der Ludwig-Maximilians-Universität München, Pettenkoferstr. 8a, 80336 München). As inclusion criteria for the study, average demographic parameters in size and weight were defined for the deceased human body. The investigated female body had a size and weight of 173 cm and 84 kg, respectively. Imaging was performed 36h *post mortem*.

Within this study, we present the first dark-field chest radiography of a deceased human body as a proof-of-principle study for the translation of X-ray dark-field imaging towards clinical application. Underlying specifications and an overview of the image acquisition procedure are given in the following. Additionally, we present a descriptive explanation of the general concept and physical mechanisms of X-ray dark-field imaging.

### X-ray grating interferometry

**Fig 1A** shows a schematic of the human X-ray dark-field thorax scanner. The scanner utilizes an imaging technique presented earlier by Pfeiffer et al. [5]. A three grating arrangement is used in combination with a conventional clinical X-ray source and an X-ray detector [5,6] in order to obtain an X-ray system that is sensitive to ultra small-angle scattering (dark-field) properties of the samples. The first grating downstream of the patient (G_1_) generates a periodic intensity pattern that can be analyzed by means of a second absorption grating (G_2_), as the pattern as such cannot be directly resolved with a clinical detector. Typically, the intensity pattern can be sampled in a step-wise manner by translating one of the gratings and acquiring images at intermediate positions. The retrieved intensity curve, also referred to as the stepping curve, yields information on the sample’s attenuation, differential phase, and ultra small-angle scattering properties with respect to X-rays. To extract the relevant signals from the measured curve, Fourier analysis or (as in our case) a least squares fit is performed. The oscillation strength of the intensity pattern normalized to its mean value, the so-called visibility, is the determining performance parameter in grating-based X-ray imaging.

Additionally, as the focal spot is large compared to the period of the intensity pattern, a third grating has to be introduced in order to shape the large source spot (1.2 mm in our case) into several partially coherent slit sources. The inter-grating distances and grating periods must fulfill the intercept theorem as described in Ref. [5]. The source to detector distance of our system measures 2010 mm.

### X-ray dark-field image contrast

Objects with strong electron density fluctuations on the micron and sub-micron scale cause ultra small-angle X-ray scattering due to repeated refraction on interfaces of the inherent micromorphology. Ultra small-angle scattering causes blurring of the intensity pattern generated by G_1_ and consequently leads to a reduction in measured visibility. The underlying mechanism is schematically illustrated in **Fig 1B**: Here X-rays are refracted at air-tissue interfaces of lung alveoli, which subsequently causes blurring (red curve) of the originally well distinguishable intensity pattern (black curve). As proposed by Pfeiffer et al. [6], this entity can be measured with a three-grating interferometer and was named—in analogy to visible light and electron microscopy—X-ray dark-field contrast.

The ultra small-angle scattering power of a certain material, which is determined by both chemical and structural properties, is described by the so-called linear diffusion coefficient [7]. Similar to the Lambert-Beer law of attenuation, the visibility shows an exponential decay with object thickness [7]. We therefore present the dark-field radiographies as the negative logarithm of the relative change in visibility to visualize the integrated lung-specific linear diffusion coefficient. Detailed considerations of X-ray dark-field contrast generation and its dependence on structural parameters can be found in Refs. [13,20]

**Fig 2:**
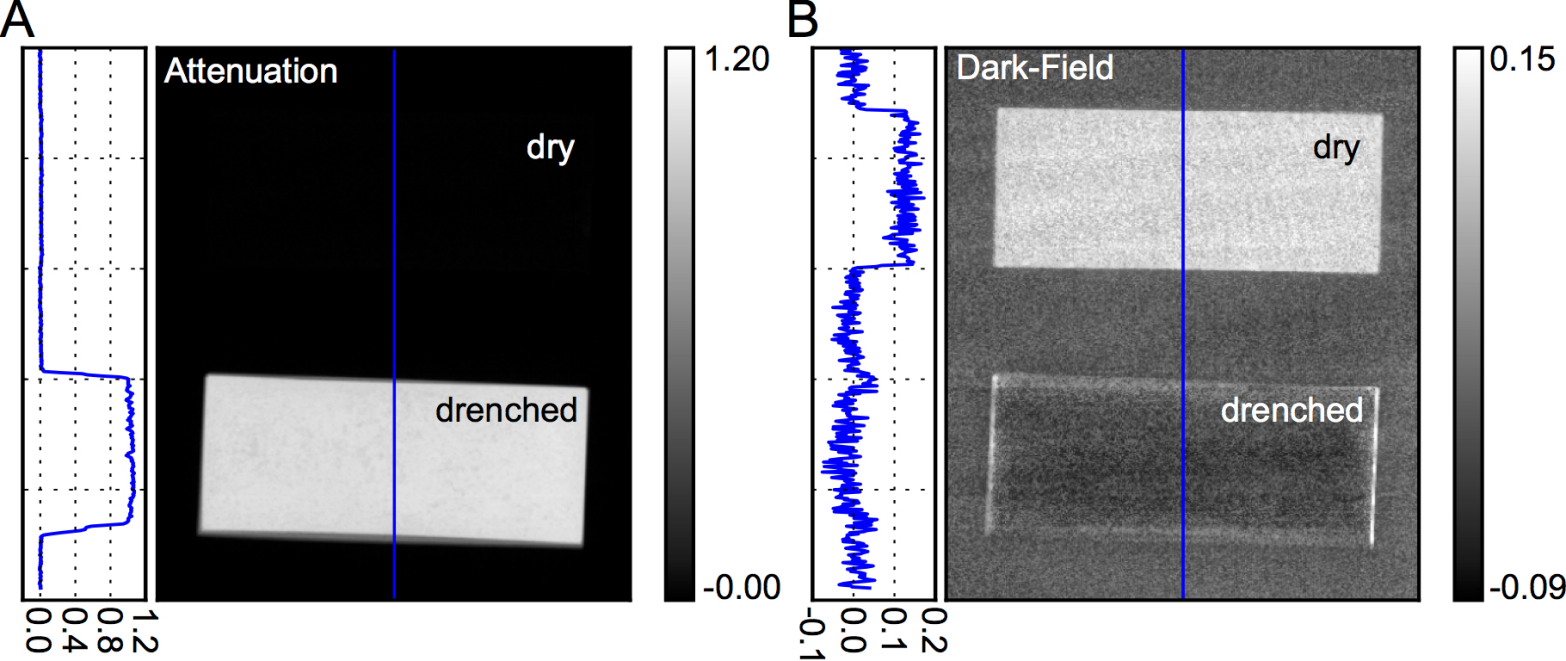
Complementarity of X-ray attenuation and dark-field radiography. X-ray attenuation (**A)** and dark-field (**B)** signal of dry (upper) and water drenched (lower) open cell melamine sponges. Similar to the human lung, a fine-pored melamine sponge comprises thousands of air-tissue interfaces and causes strong ultra small-angle X-ray scattering and therefore exhibits a distinct dark-field signal. At the same time, comprising porous structures, both lung and sponge yield small overall densities and appear radiolucent in the attenuation channel. When drenching the foam, air is displaced by water resulting in a high contrast in the attenuation channel due to stronger attenuation in comparison to air. Simultaneously, the interfaces become neutralized, explaining an inversion of both contrast modalities. The image acquisition protocol except for the tube current was similar to the one of the dead body scan (70 kVp, 100mA, 20 ms exposure time per pulse, 12 Hz pulse frequency, 3x3 pixel binning).

At present, contrast formation in clinical radiography solely relies on the attenuation properties of the investigated region of interest. In the case of the human thorax, the assessment of the lung on a radiographic basis is therefore limited, as the lung contrast is inherently low and surrounding/superimposing tissue (bones, fat, and muscle) dominate the signal. Consequently, it is only possible to identify variations in global lung pattern as such, rather than assessing its alveolar structure and potential (early) disorders of the latter. To visualize this aspect, **Fig 2** shows the attenuation and dark-field images of two open cell melamine sponges (dry and drenched with water), which were imaged with our prototype system. Similar to the human lung, a fine-pored melamine sponge consists of thousands of air-tissue interfaces which causes significant ultra small-angle X-ray scattering and therefore exhibits a distinct dark-field signal (**Fig 2B)**. At the same time, comprising porous structures, both lung and sponge yield a low overall density and appear radiolucent in the attenuation channel (**Fig 2A)**. When drenching the foam, air is displaced by water and the surfaces become wet; hence, the initial high variation of the refraction index within the sample becomes neutralized, and the dark-field signal strength decreases distinctly. Additionally, water is more attenuating in comparison to air, explaining an inversion of both contrast modalities.

This basic phantom study illustrates the potential of multimodal imaging for clinical diagnostics: by probing two different physical mechanisms, complementary image information can be retrieved. Samples that still appear radiolucent in attenuation-based imaging can be assessed by dark-field radiography via detection of ultra small-angle scattering due to inherent substructures. In studies involving the lung, this information has been demonstrated to encode physiological properties of the organ as dark-field intensity correlates well with the number of intact alveolar interfaces [21]. This motivates us to consider dark-field imaging as a functional medical imaging tool.

### Preparations

Tracheal intubation was performed on the deceased human body and the lungs were inflated with 23 mbar during measurement. This was done with the purpose of restoring ultra small-angle scatter-active interfaces by partially reverting a collapse of lung alveoli, considering measurements were conducted 36 hours after death, at which point *post-mortem* changes to the lung parenchyma are to be expected.

### Large field of view—scanning data acquisition procedure

To image an entire human thorax, the image acquisition was realized with a continuous scanning approach using an oblong, stitched grating covering an area of 2.5 x 40 cm^2^. The underlying image acquisition procedure is similar to the method proposed by Kottler et al. [22]: Instead of stepping one of the gratings between intensity recordings, the relative shifts of the intensity pattern with respect to the G_2_ grating are intrinsically encoded within the field-of-view of the interferometer. For this reason, low frequency Moiré-fringes were purposefully created by inducing a slight period mismatch between the G_1_ and the G_2_ grating. When the interferometer and the sampling pattern is then moved in relation to the specimen, its integrated X-ray interaction is recorded at various relative grating to intensity pattern positions for each pixel by consecutive intensity measurements with the detector over the course of the scan. The obtained curve corresponds to the registered intensity in a pixel as a function of the interferometer position and is equivalent to the conventional stepping curve mentioned before. Adapted to our geometry and scanning procedure, the algorithm utilized for retrieving both, attenuation and dark-field signal is similar to the one described in Ref [10]. No spatial resorting process is required prior to analyzing the stepping curve.

Since a conventional X-ray source is used, the underlying spectrum is highly polychromatic. As a consequence, the scanner is susceptible to spectral effects, so that the system visibility is dependent on sample thickness and material. Comparable considerations on this topic can be found in Ref. [23]. To account for these so-called beam-hardening effects, the relative visibility is corrected by a calibration factor previously obtained by a series of visibility measurements with an equivalent absorbing material (polyoxymethylene or POM) that mimics the spectral influences of the cadaver on the X-ray spectrum.

### Grating production and alignment

The grating structures were manufactured utilizing X-ray lithography processes [24] (KIT and microworks GmbH, Karlsruhe, Germany). In order to obtain oblong grating arrays for G_1_ and G_2_ an approach as described in [25] was utilized. Hereby, eight individual grating tiles (2.5 x 5 cm^2^) for each plane were stitched together under microscope observation. Within the scanner the individual tiles as well as assembled grating array were aligned and positioned relative to each other to fulfill the geometrical constraints indicated in **Fig 1B**. The scanner achieves a mean visibility of about 32% over the entire field-of-view.

### X-ray components and operational parameters

The X-ray source, a Philips SRO1750ROT360 (Philips Medical Systems DMC GmbH, Hamburg, Germany) is operated at 70 kVp voltage, 400 mA tube current and 12 Hz pulse frequency. That choice of acceleration voltage is a compromise between the achieved visibility and a reasonable transmission through a human thorax. With increasing X-ray energies, the visibility decreases as the gratings become more transparent. Furthermore, the spectrum becomes broader–both negatively affecting the image quality of the dark-field image. A Pixium RF 4343 (Trixell, Moirans, France) flat-panel detector with a pixel size of 148 x 148 μm^2^ is operated in a 3x3-binning mode. The exposure time per pulse was set to 20 ms. With a scan-speed of approximately 11 mm/s the entire human thorax was scanned within 30 seconds, which corresponds to 354 pulses total. Both X-ray tube and detector are standard components of medical radiographic imaging systems. The effective field of view with respect to the position of the deceased body that is achievable with these parameters and the earlier mentioned active grating area is 28 x 32 cm^2^.

### CT Image acquisition, reconstruction and radiation dose

CT imaging was performed on a 256-slice medical CT-scanner (Brilliance iCT, Philips Healthcare, Best, The Netherlands). The deceased body was placed in supine position in the gantry isocenter. Two localizer radiographs were acquired to plan the chest CT. Imaging was performed at 120 kVp, using 128*0.625 mm collimation, 0.664 pitch, 0.33 s gantry rotation time, spiral acquisition mode and Dose Right Index 5. The tube output was 28 mAs, Images were reconstructed by help of iDose4(Philips Healthcare, Best, The Netherlands). The volume computed tomography dose index was 1.9 mGy and dose line product was 76.3 mGy*cm.

### Dose considerations

The scanner was designed to be compatible with the strict dose regulations of clinical radiography. According to Wall et al.[26] the average values of effective dose deposition in posterior-anterior (PA) X-ray chest imaging is 0.014 mSv and 0.038 mSv in lateral (LAT) orientation. First, a slot collimation was implemented to limit the X-ray beam to the active grating region. Second, and in order to absorb dose-critical low-energy photons from the incident beam 2.5 mm of aluminum equivalent filtration was used. Third, by synchronizing the detector measurement with the exposure pulses, an unwanted dose deposition was avoided during signal readout. With these measures, we achieved an air-kerma of 0.54 mGy measured at the patient position. This corresponds to an effective dose value larger by a factor of 3.6 compared to a clinical PA thorax acquisition and to a similar value obtained in combined investigations (PA&LAT). This comparison was estimated by using a thorax specific air kerma conversion that was calculated by help of the conversion factors given in ICRP 103 [27] and the organ doses from Drexler et al. [28] (0.095 mSv/Gy cubic interpolated to obtain a value for 70kVp). The air kerma was measured with a PTW NOMEX dosimeter (PTW, Freiburg, Germany) combined with an air chamber sensor.

## Results

**Fig 3:**
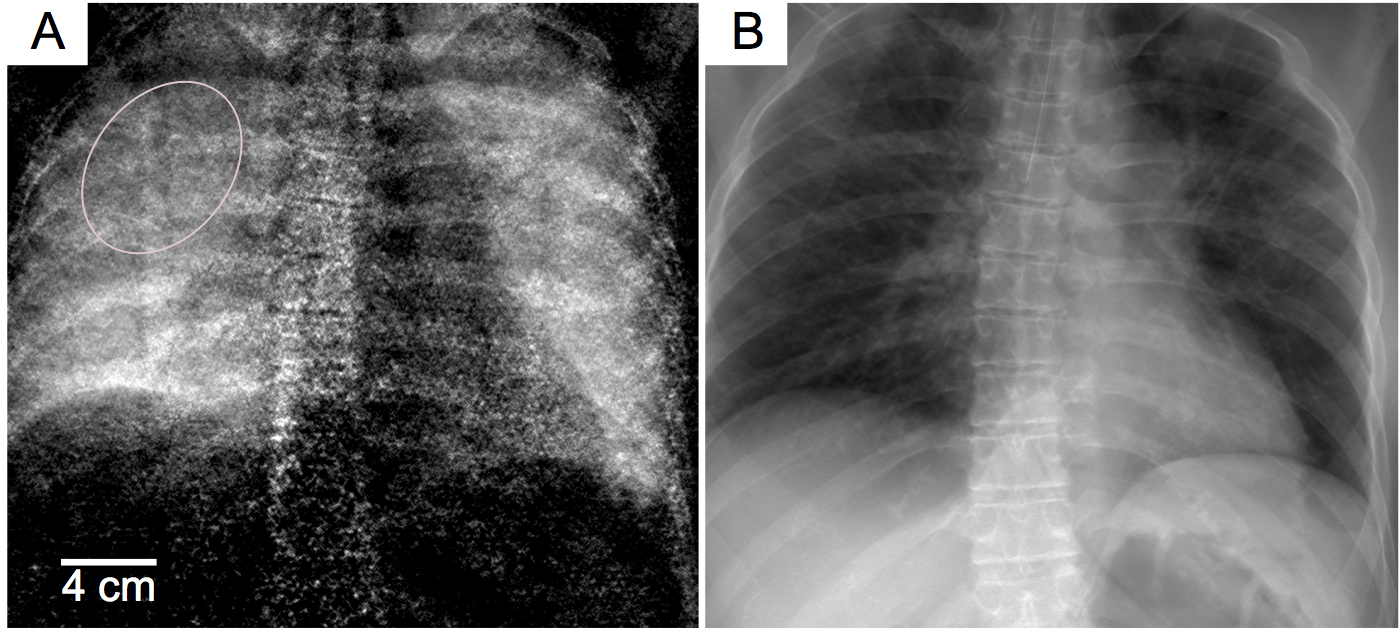
Dark-field and conventional human chest radiography images of a deceased body. Anteroposterior (AP) X-ray dark-field (**A**) and absorption (**B**) contrast images acquired with the scanner described in the materials and methods section. We measured a slight decrease in the dark-field signal intensity in the regions highlighted by the marker, indicating an edema (consistent with the findings in the autopsy and CT investigation, see **Fig 3**). This behaviour illustrates the signal formation mechanism, because the water in the lung reduces the contrast between the air-tissue interfaces, and subsequently reduces the ultra small-angle scattering (dark-field) signal.

In this study, we demonstrate for the first time the successful translation of X-ray dark-field radiography from small-animal imaging systems to a setup capable of imaging human thoraces, including imaging parameters such as scan duration, field-of-view, and radiation dose being appropriate for patients. **Fig 3** displays the dark-field and the attenuation chest images of a deceased human body. The investigated female body had a size and weight of 173 cm and 84 kg, respectively. The conventional X-ray image (**Fig 3B**) shows strong contrast between bones and soft tissue, primarily due to increased attenuation of calcium in comparison to lighter organic materials. The lungs exhibit a very low signal, as their main constituent is low attenuating air. In the dark-field channel (**Fig 3A**), a distinct pulmonary signal is measured originating from lots of high difference of the refractive index (air to tissue) along the beam path. This variation directly correlates with the microarchitecture of the lung parenchyma. Inherently, the two images are perfectly registered as their information is extracted from the same dataset recorded within one pixel. In the imaging process the interferometer is constantly scanned relative to the body’s chest while the detector acquires consecutive intensity recordings.

**Fig 4:**
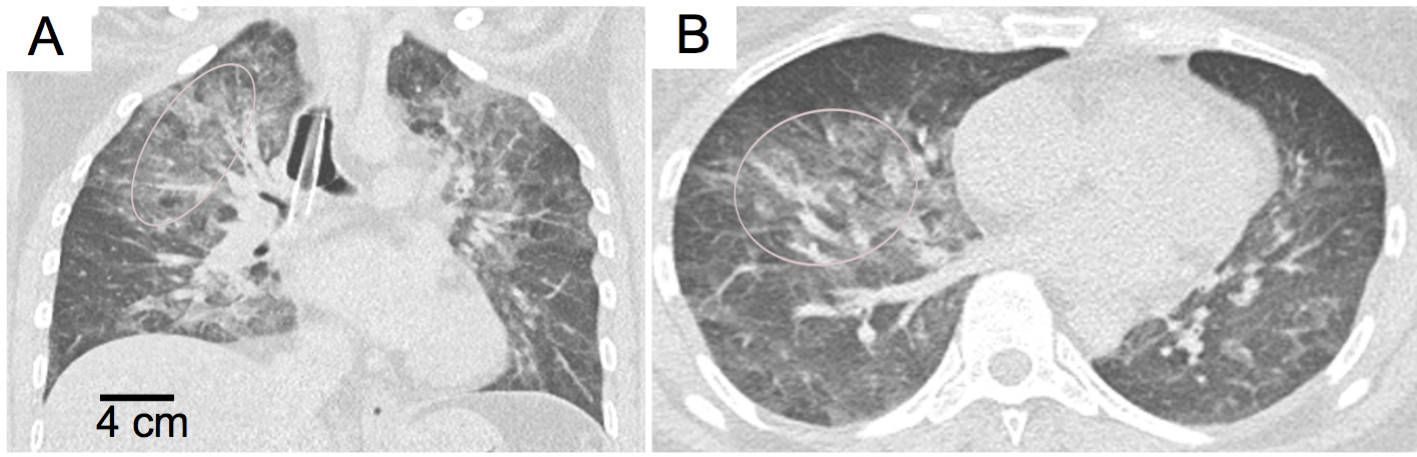
Computed tomography (CT) human chest images of a deceased body showing pulmonary edema due to cardiac failure. Coronal CT image (**A**) at a level dorsal to the tracheal bifurcation and axial CT image (**B**) of the lower lobes. The pulmonary edema indicated by the markers presents as diffuse ground-glass opacities with a predominant perihilar distribution. Both images: 3 mm slice thickness, window level -600 HU, window width 1700 HU.

For reasons of comparison and verification of our results, we conducted additional imaging with a clinical CT system (Brilliance iCT, Philips Healthcare, USA). Correlating well with CT scan results, autopsy revealed pulmonary edema due to cardiac arrest (**Fig 4**). Areas of low dark-field signal correlated with ground-glass opacities in the CT images where an accumulation of fluid reduces air-tissue-interfaces leading to a decrease of X-ray ultra small-angle scattering. In contrast, a slightly increased dark-field signal was monitored in better inflated areas of lung parenchyma. On this note, these results are obtained from a lately deceased body and an increased image quality can be expected in living subjects imaged in standing position. With respect to the observed changes in the present dark-field images, this is consistent with results obtained in mice with structural lung parenchyma changes either due to loss of pulmonary tissue (in the case of emphysema) or the replacement with fibrotic tissue (in pulmonary fibrosis). While, in contrast to pulmonary emphysema or fibrosis, the fluid accumulation in pulmonary edema does not represent permanent structural changes to the lungs, it neutralizes the air-tissue interfaces essential for the formation of the dark-field signal. The effect of fluid-filled regions in porous media has already been investigated in materials science. Yang et al. could show that in time-lapse X-ray dark-field contrast radiographs of a mortar during capillary imbibition from the bottom, the wetting front was visible due to the decrease of the dark-field signal [29].

At the current stage the preclinical data on X-ray dark-field imaging of the lungs suggests a decrease in intensity of the dark-field signal as a nonspecific but very sensitive indicator for pulmonary pathologies that affect the typical structure of air-tissue-interfaces in healthy lung parenchyma. This observation is supported by the aforementioned recent study investigating dark-field imaging in a mouse model of neonatal lung injury due to mechanical ventilation and supplementation of oxygen-enriched gas [18]. Additionally, in a mouse model of lung cancer, regions with cancerous tissue show a reduced air:tissue volume ratio due to uncontrolled cell proliferation and loss of alveolar space and therefore X-ray scattering is strongly reduced [30]. Based on previous publications in small-animal models, it is unlikely that the overall conclusions of our study are compromised by the fact that imaging was performed on a dead human body rather than living subject. As *post-mortem* changes to the lungs start immediately after death, with an attenuation gradient of ground-glass opacities in the dependant lung regions described as a typical finding, it can be assumed that the dark-field signal will be higher in living subjects. Yet, the influence of depth of inspiration on the magnitude of the X-ray dark-field signal has to be addressed in a clinical study. Our system’s current imaging time of approximately 30 seconds is long compared to exposure times of typically less than a second in clinical radiography. This is due to image acquisition being performed in a scanning mode, but can be reduced to clinically compatible exposure times with the development of gratings covering the entire field-of-view. The development of larger gratings, which cover the full field-of-view, is currently investigated and advances can be expected in the near future. In the meantime, every intermediate increase in grating size reduces the acquisition time. Further techniques used in the early days of spiral lung CT can be applied to reduce motion artefacts.

The air-kerma of 0.54 mGy was measured at the patient position. This exposure corresponds to an estimated radiation dose deposited for one X-ray dark-field image, as shown in **Fig 3,** being about four times higher compared to a standard clinical chest X-ray (PA) and roughly similar compared to combined investigations (PA&LAT). However, our setup acquires dark-field and conventional attenuation (and differential phase) images at the same time.

## Discussion

With respect to lung diagnostics, studies have estimated that 60–85% of the general population suffering from COPD, mainly those with mild to moderate symptoms, have not been diagnosed [31]. COPD is not curable; however, the substantial morbidity and mortality resulting from under-diagnosis could be prevented since COPD is a treatable disorder [32]. For example, if detected early, smoking cessation can substantially slow disease progression. Spirometry is commonly used to diagnose COPD, but it is highly dependent on the patient’s cooperation and effort. Conventional chest radiographs have poor to moderate sensitivity, particularly in patients with mild to moderate emphysema, and cannot be used to reliably assess emphysema severity. However, the presented results in a lately deceased body demonstrate that X-ray dark-field imaging of the human lung is feasible. It is obvious that X-ray dark-field radiography is not limited to COPD imaging. Its high sensitivity for visualizing the integrity of healthy lung parenchyma creates many opportunities in the field of pulmonary imaging. Yet, issues like the influence of body size/composition and breathing position on X-ray dark-field imaging have to be further investigated.

In this study, we demonstrated that X-ray dark-field radiography is capable of visualizing the dark-field signal of the lung in human sized chests. Additionally, the concept how dark-field signal strength is influenced by pathological findings in the lungs is supported by correlations with CT imaging. Further studies on deceased bodies will enable the technology to mature for living subjects and clinical application. However, large scale clinical studies will be necessary to investigate the full potential of X-ray dark-field radiography for pulmonary medicine. It will be essential to demonstrate that X-ray dark-field radiography has the potential to considerably improve lung diagnostics with a focus on COPD, allowing early detection and monitoring of the disease process.

## References

[1] WHO. The top 10 causes of death. World Heath Organisation http://www.who.int/mediacentre/factsheets/fs310/en/ (2015).

[2] Rigon, Rigon L, Besch HJ, Arfelli F, Menk RH, Heitner G, Plothow-Besch H. A new DEI algorithm capable of investigating sub-pixel structures. Journal of Physics D: Applied Physics. 2003 4 28;36(10A):A107.

[3] Endrizzi M, Olivo A. Absorption, refraction and scattering retrieval with an edge-illumination-based imaging setup. Journal of Physics D: Applied Physics. 2014 11 24;47(50):505102.

[4] Wang H, Kashyap Y, Cai B, Sawhney K. High energy X-ray phase and dark-field imaging using a random absorption mask. Scientific reports. 2016;6. 10.1038/srep30581 PMC4964655 27466217

[5] Pfeiffer F, Weitkamp T, Bunk O, David C. Phase retrieval and differential phase-contrast imaging with low-brilliance X-ray sources. Nature physics. 2006 4 1;2(4):258–61.

[6] Pfeiffer F, Bech M, Bunk O, Kraft P, Eikenberry EF, Brönnimann C, et al Hard-X-ray dark-field imaging using a grating interferometer. Nature materials. 2008 2 1;7(2):134–7. 10.1038/nmat2096 18204454

[7] Bech M, Bunk O, Donath T, Feidenhans R, David C, Pfeiffer F. Quantitative x-ray dark-field computed tomography. Physics in medicine and biology. 2010 8 31;55(18):5529 10.1088/0031-9155/55/18/017 20808030

[8] Willner M, Herzen J, Grandl S, Auweter S, Mayr D, Hipp A, et al Quantitative breast tissue characterization using grating-based x-ray phase-contrast imaging. Physics in medicine and biology. 2014 3 10;59(7):1557 10.1088/0031-9155/59/7/1557 24614413

[9] Scherer K, Willer K, Gromann L, Birnbacher L, Braig E, Grandl S, et al (2015) Toward Clinically Compatible Phase-Contrast Mammography. PLoS ONE 10(6): e0130776 10.1371/journal.pone.0130776 26110618 PMC4481352

[10] Koehler T, Daerr H, Martens G, Kuhn N, Löscher S, Stevendaal U, et al Slit‐scanning differential x‐ray phase‐contrast mammography: Proof‐of‐concept experimental studies. Medical physics. 2015 4 1;42(4):1959–65 10.1118/1.4914420 25832086

[11] Momose A, Yashiro W, Kido K, Kiyohara J, Makifuchi C, Ito T, et al X-ray phase imaging: from synchrotron to hospital. Phil. Trans. R. Soc. A. 2014 3 6;372(2010):20130023 10.1098/rsta.2013.0023 24470409 PMC3900032

[12] Bech M, Tapfer A, Velroyen A, Yaroshenko A, Pauwels B, Hostens J, et al In-vivo dark-field and phase-contrast x-ray imaging. Scientific reports. 2013 11 13;3:3209 10.1038/srep03209 24220606 PMC3826096

[13] Yashiro W, Terui Y, Kawabata K, Momose A. On the origin of visibility contrast in x-ray Talbot interferometry. Optics express. 2010 8 2;18(16):16890–901 10.1364/OE.18.016890 20721081

[14] Meinel FG, Yaroshenko A, Hellbach K, Bech M, Müller M, Velroyen A, et al Improved diagnosis of pulmonary emphysema using in vivo dark-field radiography. Investigative radiology. 2014 10 1;49(10):653–8 10.1097/RLI.0000000000000067 24853070

[15] Hellbach K, Yaroshenko A, Meinel FG, Yildirim AÖ, Conlon TM, Bech M, et al In vivo dark-field radiography for early diagnosis and staging of pulmonary emphysema. Investigative radiology. 2015 7 1;50(7):430–5 10.1097/RLI.0000000000000147 25761095

[16] Yaroshenko A, Hellbach K, Yildirim AÖ, Conlon TM, Fernandez IE, Bech M, et al Improved in vivo assessment of pulmonary fibrosis in mice using x-ray dark-field radiography. Scientific reports. 2015 12 1;5:17492 10.1038/srep17492 26619958 PMC4664921

[17] Hellbach K, Yaroshenko A, Willer K, Pritzke T, Baumann A, Hesse N, et al Facilitated diagnosis of pneumothoraces in newborn mice using x-ray dark-field radiography. Investigative radiology. 2016 10 1;51(10):597–601 10.1097/RLI.0000000000000285 27603110

[18] Yaroshenko A, Pritzke T, Koschlig M, Kamgari N, Willer K, Gromann L, et al Visualization of neonatal lung injury associated with mechanical ventilation using x-ray dark-field radiography. Scientific reports. 2016 4 13;6:24269 10.1038/srep24269 27072871 PMC4829826

[19] Gromann LB, De Marco F, Willer K, Noël PB, Scherer K, Renger B, et al In-vivo x-ray dark-field chest radiography of a pig. Scientific Reports. 2017 7 6;7(1):4807 10.1038/s41598-017-05101-w 28684858 PMC5500502

[20] Malecki A, Potdevin G, Pfeiffer F. Quantitative wave-optical numerical analysis of the dark-field signal in grating-based x-ray interferometry. EPL (Europhysics Letters). 2012 8 17;99(4):48001.

[21] Yaroshenko A, Meinel FG, Bech M, Tapfer A, Velroyen A, Schleede S, et al Pulmonary emphysema diagnosis with a preclinical small-animal x-ray dark-field scatter-contrast scanner. Radiology. 2013 11;269(2):427–33 10.1148/radiol.13122413 23696682

[22] Kottler C, Pfeiffer F, Bunk O, Grünzweig C, David C. Grating interferometer based scanning setup for hard x-ray phase contrast imaging. Review of Scientific Instruments. 2007 4;78(4):043710 10.1063/1.2723064 17477673

[23] Chabior M, Donath T, David C, Bunk O, Schuster M, Schroer C, et al Beam hardening effects in grating‐based x‐ray phase‐contrast imaging. Medical physics. 2011 3 1;38(3):1189–95 10.1118/1.3553408 21520831

[24] Mohr J, Grund T, Kunka D, Kenntner J, Leuthold J, Meiser J, Schulz J, Walter M. High aspect ratio gratings for x-ray phase contrast imaging. InAIP Conference Proceedings 2012 7 31 (Vol. 1466, No. 1, pp. 41–50). AIP.

[25] Schröter TJ, Koch FJ, Meyer P, Kunka D, Meiser J, Willer K, et al Large field-of-view tiled grating structures for X-ray phase-contrast imaging. Review of Scientific Instruments. 2017 1;88(1):015104 10.1063/1.4973632 28147659

[26] Wall BF, Haylock R, Jansen JT, Hillier MC, Hart D, Shrimpton PC. Radiation risks from medical X-ray examinations as a function of the age and sex of the patient. Health Protection Agency Centre for Radiation, Chemical and Environmental Hazards HPA-CRCE-028. 2011

[27] ICRP 103 The 2007 Recommendations of the International Commission on Radiological Protection. Ann- ICRP.2007;37(2.4):2. 10.1016/j.icrp.2007.10.003 18082557

[28] Drexler G. Die Bestimmung von Organdosen in der Röntgendiagnostik. Hoffmann; 1993

[29] Yang F, Prade F, Griffa M, Jerjen I, Di Bella C, Herzen J, Sarapata A, Pfeiffer F and Lura P. (2014). „Dark-field X-ray imaging of unsaturated water transport in porous materials”, Applied Physics Letters 105 (15), 154105 (5 pp.)

[30] Scherer K, Yaroshenko A, Bölükbas DA, Gromann LB, Hellbach K, Meinel FG, Braunagel M, Berg JV, Eickelberg O, Reiser MF, Pfeiffer F, Meiners S, Herzen J. X-ray Dark-field Radiography—In-Vivo Diagnosis of Lung Cancer in Mice. Sci Rep. 2017 3 24;7(1):402 10.1038/s41598-017-00489-x 28341830 PMC5428469

[31] Halbert RJ, Isonaka S, George D, Iqbal A. Interpreting COPD prevalence estimates: what is the true burden of disease?. Chest journal. 2003 5 1;123(5):1684–92. 10.1378/chest.123.5.1684 12740290

[32] Mannino DM, Buist AS. Global burden of COPD: risk factors, prevalence, and future trends. The Lancet. 2007 9 7;370(9589):765–73 10.1016/S0140-6736(07)61380-4 17765526

